# The Four Deadly Sins of Implicit Attitude Research

**DOI:** 10.3389/fpsyg.2020.604340

**Published:** 2021-01-18

**Authors:** Jeffrey W. Sherman, Samuel A. W. Klein

**Affiliations:** Department of Psychology, University of California, Davis, Davis, CA, United States

**Keywords:** implicit attitudes, bias, modeling, automaticity, control

## Abstract

In this article, we describe four theoretical and methodological problems that have impeded implicit attitude research and the popular understanding of its findings. The problems all revolve around assumptions made about the relationships among measures (indirect vs. versus direct), constructs (implicit vs. explicit attitudes), cognitive processes (e.g., associative vs. propositional), and features of processing (automatic vs. controlled). These assumptions have confused our understandings of exactly what we are measuring, the processes that produce implicit evaluations, the meaning of differences in implicit evaluations across people and contexts, the meaning of changes in implicit evaluations in response to intervention, and how implicit evaluations predict behavior. We describe formal modeling as one means to address these problems, and provide illustrative examples. Clarifying these issues has important implications for our understanding of who has particular implicit evaluations and why, when those evaluations are likely to be particularly problematic, how we might best try to change them, and what interventions are best suited to minimize the effects of implicit evaluations on behavior.

## Introduction

In this article, we describe four long-standing theoretical and methodological problems that have hindered understanding of implicit evaluations. With tongue planted firmly in cheek, we refer to these as the “deadly sins” of implicit attitude research. To some extent, everyone working in the field has participated in the promotion of these ideas at one point or another, ourselves included. However, we have now reached a point at which all researchers of implicit attitudes should understand these problems and actively seek to avoid making them. The consequences are clear. When we commit these “sins,” we undermine our ability to understand exactly what it is we are measuring, what it means when implicit evaluations differ across people or contexts or when they change, and when and how implicit evaluations predict behavior. These are not merely academic concerns. How we understand and characterize implicit evaluation has direct implications for a range of critical pragmatic issues, including assigning responsibility for possessing or altering problematic implicit evaluations, understanding when implicit evaluations are likely to be most problematic, and designing interventions that effectively alter implicit evaluations and its influence on broader behavior.

## Definitional Issues

### Indirect Versus Implicit Measures

Let us first settle some definitional issues. We refer to measures of implicit evaluation as “indirect measures.” We use the term “indirect” rather than “implicit” because “implicit” implies features of automaticity that the measures may or may not possess. For example, responses may or may not be fast and efficient, respondents may or may not be aware of the purpose of the measure, may or may not intend particular outcomes on the measure, and may or may not be able to intentionally alter responses on the measure. Indeed, the plethora of indirect measures differ substantially along these dimensions. The only feature that they all share is that they infer evaluations from performance on some task rather than by directly asking respondents to provide them (e.g., Corneille and Hütter, 2020; Gawronski et al., 2020). That is, they are indirect measures of evaluation.

The same problems apply to understanding the outcomes of indirect measures. These evaluations may or may not operate efficiently, may or may not have been formed intentionally, may or may not be controlled, and respondents may or may not be aware of their content. Which of these features apply is dependent on the means by which the evaluation is measured (i.e., which indirect measure is used) and the subject of the evaluation (e.g., race, age, fruit, dogs, etc.), among many factors. For these reasons, we believe that implicit evaluations are more accurately described as indirect evaluations. Nevertheless, given the prominence of the term “implicit” when describing evaluations, both in academic and popular culture, such a change is simply too impractical. But, note that our use of the term “implicit evaluation” signifies only that the evaluations are implied by performance on an indirect measure rather than explicitly provided on a direct measure. In that sense, the evaluations are, indeed, implicit in the given responses. However, the term does not imply features of automaticity that often are ascribed to these evaluations.

### Evaluations Versus Attitudes

We use the term “evaluation” rather than “attitude,” except where it would violate normative usage in ways that may confuse the reader (e.g., in describing the broad field of implicit attitude research). The term “evaluation” recognizes the constructive nature of responses on indirect measures. A measure is not a direct and pure reflection of an evaluative mental representation that exists in the mind waiting to be discovered. Rather, a variety of processes interact to produce a response on each trial of the measure, such as accurately detecting a correct response, inhibiting incorrect responses, response biases, and many more (e.g., Sherman et al., 2010; Hütter and Klauer, 2016; Calanchini et al., 2018). Moreover, as we will detail below, those processes intervening between stimulus and response can be identified and measured. Thus, the responses reflect not Things that we have (e.g., Eagly and Chaiken, 2007; Fazio, 2007; Petty et al., 2007), but evaluative behaviors we enact (e.g., Schwarz, 2007). Of course, there is information in memory that contributes to these evaluative responses, but the outcomes of the measures are far from direct indicators of a latent, stable, Attitude Thing.

Another negative consequence of the Attitude Thing view is that it creates unrealistic expectations of cross-situational consistency and temporal stability in implicit evaluations. If indirect measures reveal Things that are stored in memory, then those Things might be expected to demonstrate a fair degree of consistency and stability. However, counter to early theorizing, it is now clear that implicit evaluations show both considerable context specificity and malleability (e.g., Gawronski and Brannon, 2019). This suggests that the evaluations resulting from indirect measures are constructed as needed and may be based on a variety of knowledge and processes that vary in accessibility across place and time.

A related implication of this view is that the implicit Attitude Things assessed with different indirect measures ought to correlate strongly with one another, presuming the measures are all tapping the same underlying Thing. However, implicit evaluations assessed with different measures correspond modestly, at best (Bar-Anan and Nosek, 2014). This suggests that different measures activate different information in memory and invoke different processes in responding. In turn, this suggests that the outcomes of indirect measures are more accurately viewed as evaluations than as stable attitudes.

## The Deadly Sins

### Dual Process Origins

In terms of a broad theoretical orientation, the source of much complication (the “original sin” as it were) is the grounding of implicit attitude research in dual process models of psychology that distinguish between automatic and controlled classes of processes. Whereas automatic processes occur without awareness or intention, cannot be controlled, and are highly efficient, controlled processes operate with awareness and intention, can be controlled, and require cognitive resources (Bargh, 1994). Though they have been tremendously successful and influential (Sherman et al., 2014a), dual process models have left us with some unfortunate theoretical baggage. In framing implicit (versus explicit) attitudes, that baggage consists of the general notion that there are two qualitatively distinct types of processes (e.g., Fazio et al., 1995; Greenwald et al., 1998; Strack and Deutsch, 2004; Gawronski and Bodenhausen, 2006) and/or mental representations (e.g., Greenwald et al., 1998; Wilson et al., 2000) and/or mental systems (e.g., Rydell and McConnell, 2006) corresponding to qualitatively distinct implicit and explicit attitudes. According to this mapping, implicit and explicit attitudes are reflective of distinct types of processes (e.g., spreading activation versus executive functions) that operate under distinct conditions (e.g., when control is and is not possible) and that are based on distinct types of mental representations (e.g., associations versus propositions). These assumptions have not held up to scrutiny and are responsible for the four deadly sins (for reviews, see Sherman et al., 2014b; Gawronski and Hahn, 2019).

### Deadly Sin #1: Conflating Measures and Constructs

The first and most fundamental sin is the confounding of measures and constructs (Table 1). The operational manifestation of the basic claim that implicit and explicit attitudes are distinct constructs rests on the assumption that responses on indirect and direct measures reflect distinct processes and/or mental representations and/or systems that operate under distinct conditions. However, the data do not support such clear distinctions. Indirect and direct measures are not easily divided into measures that reflect automatic versus controlled processing, invoke spreading activation versus executive processes, or call upon associative versus propositional knowledge. Moreover, there is considerable variation among indirect measures (and among direct measures) along these dimensions, calling into question the notion that there are coherent categories of indirect (and direct) measures that all possess certain features, beyond being indirect (or direct). We will delve more deeply into these issues below.

**TABLE 1:** The four deadly sins of implicit attitude research.

Deadly Sins (conflating…)	Assumption	Example	Concern
Measures and constructs	Processes, knowledge, and conditions underlying implicit evaluations are distinct from those underlying explicit evaluations	Indirect measures reflect implicit evaluations and direct measures reflect explicit evaluations	There is considerable variation within and between measurement types; the constructs cannot be made so cleanly distinct
Operating conditions and Principles	What the process does implies the conditions under which it operates and vice versa	Assuming a process is associative because it is unaffected by cognitive load	Operating principles (e.g., inhibition) may possess both automatic and controlled features of automaticity
Measures and operating conditions	Processes underlying implicit evaluations occur under different conditions than those underlying explicit evaluations	Participants are unaware of the mental content applied to responses on an IAT	Operating conditions are confounded with task differences; empirical evidence contradicts many such claims
Measures and operating principles	Processes underlying implicit evaluations are mechanistically distinct from those underlying explicit evaluations	Using an indirect measure (e.g., SMT) to assess the activation of stereotypic associations and a direct measure (e.g., Modern Racism Scale) to assess how those activated associations are applied	Operating principles are confounded with task differences; responses are driven by more than a single process

Indirect and direct measures also differ from one another along many dimensions that are unrelated to features of automaticity/control. For example, indirect measures are more likely to use images as stimuli than are direct measures. As well, indirect measures are more likely to use individual category exemplars (e.g., specific category members) as stimuli, whereas direct measures typically refer to social categories as a whole (e.g., Black vs. White people). As such, observed differences between indirect and direct measures may reflect such structural properties and have little or nothing to do with dual process conceptions of automaticity and control. There also is considerable variation among indirect (and direct) measures on such theoretically irrelevant features. For example, whereas evaluative priming measures of implicit evaluation almost always use pictures of individuals as stimuli, the IAT sometimes uses category labels (e.g., Black vs. White).

### Deadly Sin #2: Conflating Operating Principles With Operating Conditions

The second deadly sin occurs when researchers conflate operating principles and operating conditions (Sherman, 2006; Gawronski and Bodenhausen, 2009; Gawronski et al., 2014; Sherman et al., 2014b). Operating principles refer to the qualitative nature of the cognitive processes and representations that translate inputs into outputs. That is, they describe what the process does and on what mental representations it operates (e.g., activation of associations; information integration; inhibition; propositional reasoning). In contrast, operating conditions refer to the conditions under which a given process operates (e.g., Does it operate with awareness, intention, efficiency, and/or controllability?).

Indirect measures and implicit evaluations have been interpreted to reflect both operating principles (e.g., Fazio et al., 1995; Greenwald et al., 1998; Strack and Deutsch, 2004; Gawronski and Bodenhausen, 2006) and operating conditions (e.g., Fazio et al., 1995; Greenwald et al., 1998; De Houwer et al., 2009). Problems arise when operating principles and conditions are conflated with one another. Sometimes knowledge of operating conditions leads to inferences about operating principles. For example, it has commonly been assumed that, whereas associative processes operate relatively automatically, non-associative processes (e.g., self-regulatory processes; propositional reasoning) require cognitive resources. As such, evidence that a process is unaffected by cognitive load may be taken as evidence that the process must be associative in nature (i.e., reflects the activation of associations stored in memory). In turn, researchers often infer operating conditions from knowledge about operating principles. Thus, if responses on a measure are determined to be associative in nature, it may be taken as evidence that the process must operate in an automatic fashion (e.g., Fazio et al., 1995; Strack and Deutsch, 2004; Gawronski and Bodenhausen, 2006). However, there is now ample evidence that self-regulatory processes (e.g., Glaser and Kihlstrom, 2006; Calanchini and Sherman, 2013; Moskowitz, 2014) and propositional reasoning (e.g., De Houwer, 2014) possess features of automaticity. There also is growing evidence that associative processes do not always operate automatically (e.g., De Houwer, 2014).

Thus, operating principles cannot be inferred from knowledge about operating conditions or vice versa. If one wishes to make claims about operating principles and conditions, each must be independently verified with empirical research. For example, claims that a process is self-regulatory in nature must be based on independent evidence for the operation of self-regulation. Likewise, claims that a process occurs without intention must be based on evidence regarding intentionality (Bargh, 1994; De Houwer et al., 2009; Sherman et al., 2014b).

### Deadly Sin #3: Conflating Measures With Operating Conditions

The third deadly sin refers to the common tendency to presume that indirect and direct measures reflect different operating conditions. Specifically, whereas indirect measures have been thought to reflect automatic processes, direct measures have been thought to reflect controlled processes (Devine, 1989; Fazio et al., 1995; for a review, see De Houwer et al., 2009). In this case, the measures are presumed to impose features of automaticity/control on evaluative responses.

As alluded to above, one problem with this assumption is that indirect and direct measures differ from one another along many dimensions that are theoretically unrelated to the automaticity/control distinction (e.g., the use of images versus category labels as stimuli). As a result, differences attributed to varying operating conditions may instead reflect other features of indirect and direct measures. There can be significant cost to making such an error. An instructional example can be found in the implicit memory literature. For many years, indirect measures of memory were assumed to reflect the automatic influence of memories, whereas direct measures of memory were assumed to reflect the intentional use of memory. When Roediger (1990) observed that performance on indirect measures of memory depended largely on the perceptual features of stimuli, whereas performance on direct measures of memory depended on the conceptual (meaning) features of stimuli, a large body of research was immediately open to reinterpretation.

Empirically, the assumption that indirect and direct measures map onto the automaticity/control distinction is also problematic (for a review, see De Houwer et al., 2009). For example, there is considerable evidence that respondents are aware of their implicit evaluations and how they influence task performance (e.g., Hahn et al., 2014; Gawronski and Brannon, 2019). There also is evidence that respondents can intentionally influence outcomes on indirect measures (e.g., Gawronski, 2009) and can inhibit unwanted responses (e.g., Glaser and Knowles, 2008; Sherman et al., 2008; Moskowitz and Li, 2011; Krieglmeyer and Sherman, 2012). Finally, there is considerable evidence that responses on indirect measures are influenced by the availability of processing resources, suggesting that they do not reflect entirely automatic processes (e.g., Correll et al., 2002; Conrey et al., 2005; Krieglmeyer and Sherman, 2012). Thus, it is clear that indirect measures do not necessarily constrain controlled processing. At the same time, any suggestion that automatic processes have no influence on direct measures is clearly indefensible, as, by definition, automatic processes should always be operating in process-relevant contexts.

It also is clear that there is no universal profile of operating conditions that holds across the many different indirect measures of evaluation, which correlate only modestly with one another (Bar-Anan and Nosek, 2014; Rivers et al., 2017). Different measures possess different features of automaticity and control, which would, ideally, be independently determined for each measure (e.g., De Houwer et al., 2009). The same is true of direct measures. Thus, blanket assumptions that indirect and direct measures are distinguished by a consistent set of different operating conditions are untenable.

The problem of confounding measurement type and operating conditions is compounded when operating conditions also are conflated with operating principles (the second sin). In this case, to the extent that responses on a direct measure are presumed to reflect controlled processes, they also will be presumed to reflect propositional or self-regulatory processes, but not associative processes. Thus, if two people differ on explicit evaluations, they will be assumed to differ on controlled processes that are, by definition, self-regulatory or propositional (but not associative). Likewise, to the extent that responses on an indirect measure are presumed to reflect automatic processes, they also will be presumed to reflect associative but not propositional or self-regulatory processes. Thus, if two people differ on implicit evaluations, they will be assumed to differ on automatic processes that are, by definition, associative in nature.

In this way, these confounds constrain the available explanations for accounting for differences in evaluations among people, across situations, and over time. As an example, observed age-based differences in the extent of implicit evaluative bias, by this logic, must be based on differences in automatic processes, which are, by definition, associative in nature. However, in this case, our own research has shown that age-based differences in implicit evaluative bias are associated not with differences in associative processes, but with differences in self-regulatory processes that depend on executive function (e.g., Gonsalkorale et al., 2009a, 2014). Thus, assumptions about measures and their operating conditions can inhibit our ability to accurately identify what accounts for differences in implicit evaluations among people, across contexts, over time, and in their capacity to predict behavior. Once again, the conclusion is that, if one wishes to make claims about operating conditions, then those claims must be independently verified with direct research.

### Deadly Sin #4: Conflating Measures With Operating Principles

The fourth sin refers to the common tendency to presume that direct and indirect measures reflect different operating principles (i.e., the operation of distinct mental representations or processes). Specifically, whereas indirect measures have been thought to reflect the operation of associative processes, explicit measures have been thought to reflect propositional and/or self-regulatory processes (e.g., Fazio et al., 1995; Strack and Deutsch, 2004; Gawronski and Bodenhausen, 2006). In its most extreme (and most common) form, researchers have assumed a one-to-one relationship between measures and processes; that is, that indirect and direct measures are pure measures of associative versus propositional/self-regulatory processes that reflect those and no other processes.

Just as is the case with conflating measures with constructs or operating conditions, the problem is that the different measures differ in multiple ways, some of which may be relevant, expected, and intended, and others that may be irrelevant, unexpected, and unwanted. As the measures differ in many structural features (e.g., the use of category exemplars vs. category labels), so, too, do they differ in terms of the operating principles that determine responses. Consequently, it is impossible to build a clear understanding of operating principles based on untested assumptions about which principles underlie which measures.

An additional problem with this approach is that no measure is process-pure. Not only is it complicated to infer operating principles from task performance, but each task engages multiple processes/operating principles. Though indirect measures are largely treated strictly as measures of associative processes, it is now clear that they reflect a variety of additional processes, including the inhibition of associative biases (Bartholow et al., 2006; Stahl and Degner, 2007; Sherman et al., 2008; Moskowitz and Li, 2011), the detection of appropriate responses (Payne, 2001; Correll et al., 2002; Amodio et al., 2004; Klauer et al., 2007; Sherman et al., 2008; Krieglmeyer and Sherman, 2012; Meissner and Rothermund, 2013), response biases (e.g., Klauer et al., 2007; Stahl and Degner, 2007; Sherman et al., 2008; Krieglmeyer and Sherman, 2012), bias correction processes (e.g., Krieglmeyer and Sherman, 2012), stimulus recoding (e.g., Rothermund and Wentura, 2004; Kinoshita and Peek-O’Leary, 2005; Chang and Mitchell, 2011; Meissner and Rothermund, 2013), misattribution processes (Payne et al., 2005; Payne et al., 2010), task-set shifts and task-set simplification (Mierke and Klauer, 2001, 2003), and speed-accuracy tradeoffs (e.g., Brendl et al., 2001; Klauer et al., 2007). Thus, outcomes on any indirect (and direct) measure reflect the ongoing interplay of a variety of cognitive processes, and those outcomes cannot, on their own, reveal the nature of the underlying processes that produced the outcomes.

To provide a concrete example, consider the Stroop task (Stroop, 1935). A young child who knows colors but does not know how to read will likely perform very well on the task, making few errors. An adult with full reading ability may achieve the same level of success. However, these performances would be based on very different underlying processes. In the case of the adult, the automatic habit to read the word must be overcome in order to report the color of the ink accurately on incompatible trials (e.g., the word “blue” written in red ink). In contrast, the child has no automatic habit to overcome—they only see the color of the ink. The same logic applies to many indirect measures of evaluation (which often employ the same compatibility logic as the Stroop task). For example, in an IAT on attitudes toward age, activated evaluative associations between old age and negativity may need to be overcome on incompatible trials that require participants to pair old age and positive stimuli. As such, the identical responses of two individuals may reflect mildly biased associations in one case, but strong associations that are successfully overcome in the other (e.g., Gonsalkorale et al., 2009a, 2014). Thus, the observed outcomes on indirect measures can conceal differences in underlying attitudes/associations.

Consideration of the Stroop and IAT tasks illustrates the problem in another way, as well. The Stroop and the IAT are both response-conflict measures: two competing responses are simultaneously active on incompatible trials, and the conflict must be resolved in order to provide the correct response. Despite the structural similarity between these two tasks, they have been interpreted in very different ways. Whereas the Stroop task is used almost exclusively as a measure of executive function/cognitive control, the IAT is used almost exclusively as a measure of the automatic activation of associations. Of course, both conclusions are wrong. Performance on the Stroop varies as a function of language knowledge: The impulse to read a word written in English is much stronger for a native English speaker than it is for people for whom English is a second language (e.g., Tzelgov et al., 1990). Thus, performance on the Stroop reflects both the strength of the reading habit and the ability to overcome that habit when necessary. Likewise, the IAT reflects both the strength of implicit evaluative bias and the respondent’s ability to overcome that bias when necessary.

All of these problems are further exacerbated by the fact that the very same measure may recruit different processes, depending on the testing conditions. Rivers et al. (2017) have shown that evaluative implicit biases on the Stereotype Misperception Task (Krieglmeyer and Sherman, 2012), may be driven by either misattribution processes or response conflict processes, depending on experimental details. For example, when judgment targets are relatively ambiguous, misattribution processes carry more weight in driving evaluations, whereas response conflict processes play a larger role when judgment targets are relatively unambiguous. In addition, when the time between the prime and target is very brief, misattribution processes are more influential, whereas longer time delays increase the influence of response conflict processes. All of this indicates the need for tools to assess operating principles more directly.

Finally, a related drawback to conflating measures and operating principles is that, when separate measures are used to index different processes, it is impossible to examine the simultaneous contributions of those processes and how they interact with and constrain one another. With this approach, within any measure, one may examine only a single process at a time, with no means to assess the ongoing interplay of multiple processes in producing a discreet response on a particular task.

## Process Modeling to Identify Operating Principles

Here, we briefly outline one increasingly common means for identifying operating principles—the use of formal mathematical models (for more extensive reviews, see Sherman et al., 2010; Hütter and Klauer, 2016; Calanchini et al., 2018). Modeling provides means of determining which processes (operating principles) best characterize performance on a given task, the extent of those processes, and how they interact and constrain one another in producing responses. To do so, models attempt to describe outcomes on the measures (error rates, reaction times) via a set of variables (or parameters) and a set of equations that establish relationships among the variables. The variables in the equations represent the hypothesized component processes/operating principles (e.g., activation of associations, detecting a correct response, overcoming bias, response bias, etc.). Solving for these variables yields estimates of the extent of the processes. In some cases, such as with Signal Detection Theory (Green and Swets, 1966; Correll et al., 2002) or Process Dissociation (Jacoby, 1991; Payne, 2001), the equations can be solved algebraically. In other cases, such as with multinomial models (e.g., Batchelder and Riefer, 1999; Sherman et al., 2008) or diffusion models (e.g., Ratcliff, 1978; Klauer et al., 2007), parameter estimates are systematically varied through maximum likelihood estimation or related procedures to determine the values that most closely reproduce actual task performance. Process models are constrained to certain types of data. Whereas multinomial, process dissociation, and signal detection models require the input of discrete data (e.g., error rates), diffusion models also require continuous data (e.g., response times).

There are two main purposes of modeling. First, it is used to identify the processes that best account for performance on the task of interest and how those processes interact with one another. This can be achieved by comparing model fit across candidate models. Second, modeling is used to estimate the extents of the component processes. For example, the Quad model (e.g., Conrey et al., 2005; Sherman et al., 2008) estimates four processes: Association Activation, Detection, Overcoming Bias, and Guessing. Applying the model yields estimates of the extent to which each of these processes is contributing to responses.

Formal modeling offers a number of important advantages for identifying operating principles. First, because models are fit to data generated by a single task, observed differences in process estimates cannot be attributed to differences in operating conditions, operating principles, or irrelevant structural features (e.g., the use of faces versus words as stimuli) across different tasks. When, for example, estimating association activation and inhibition with two different tasks, such confounds always loom. However, estimating those processes from performance on a single task eliminates such concerns. Second and related, inherent in the use of formal models is the assumption that multiple processes interact to drive outcomes. Measures are not assumed to be process-pure. Third, specifying a model requires the development and use of an explicit theory about which processes contribute to performance and the manner in which those processes interact with one another. The development of explicit theories drives progress in understanding implicit evaluation. Finally, competing models that identify different processes or different relationships among the processes can be compared in terms of their ability to fit the data. This provides a means of comparing the validity of different theories.

### Validating Operating Principles and Conditions

Above, we described the problems with making assumptions about the operating principles and conditions of indirect measures. The same issues apply in modeling. Thus, the operating principles of model parameters must be established independently via validation studies. If a parameter is meant to reflect a self-regulatory process, then the parameter must be shown to respond the way self-regulatory efforts should. For example, if the parameter correlates with known measures of self-regulation, predicts self-regulatory behavior, is reduced when self-regulation is constrained, etc., then we can be confident that the parameter captures self-regulation. Likewise, claims about operating conditions must be independently validated. If we want to claim that a parameter is dependent on the availability of cognitive resources, then we need to show that empirically. For example, showing that a parameter is affected by a cognitive load or a short response deadline (i.e., the efficiency component of automaticity) would provide critical validation.

### Application to Fundamental Questions About Implicit Evaluation

#### What Mechanisms Produce Implicit Evaluations?

The modeling of indirect measures has played a significant role in answering fundamental questions about implicit evaluation. Most basically, modeling has shed considerable light on the question of exactly what indirect measures are measuring. Most commonly, they have been described as measuring associative processing that is reflective of evaluative associations in memory. However, in addition to associative processes, a variety of non-associative processes have been proposed as integral to responding. Often, these proposals were explicitly tested and supported via formal modeling. For example, the inhibition of associations (Sherman et al., 2008), detection of appropriate responses (Payne, 2001; Correll et al., 2002; Klauer et al., 2007; Stahl and Degner, 2007; Sherman et al., 2008; Krieglmeyer and Sherman, 2012; Meissner and Rothermund, 2013), response biases (Correll et al., 2002; Klauer et al., 2007; Stahl and Degner, 2007; Sherman et al., 2008; Krieglmeyer and Sherman, 2012), bias correction processes (Krieglmeyer and Sherman, 2012), stimulus recoding (e.g., Meissner and Rothermund, 2013), misattribution processes (Payne et al., 2010), and speed-accuracy trade-offs (e.g., Klauer et al., 2007) were all established as critical components of indirect task performance through the development and use of formal models.

At least in some cases, these processes are not even directly related to the attitude object in question. Calanchini et al. (2014), derived parameter estimates from the Quad model on IATs measuring implicit evaluations of a variety of different social and non-social categories. They examined the extent to which the parameters correlated with themselves across pairs of categories. The pairs of categories varied in the extent to which they represented similar targets and judgment attributes. For example, evaluative White/Black and White/Asian IATs both measure evaluative responses to racial groups. In contrast, evaluative White/Black and Flower/Insect IATs measure evaluative responses to very different types of categories. As expected, the parameter representing activated associations (AC) correlated across two different IATs to the extent that the IATs measured evaluations of similar categories. For example, the AC correlation between White/Black and White/Asian was stronger than the AC correlation between White/Black and Flower/Insect. As such, AC seems to represent evaluative associations that are specific to the attitude target in question. However, the parameters representing the detection of correct responses (D) and overcoming bias (OB) correlated strongly across attitude domains, regardless of conceptual overlap. In this case, the D and OB correlations between White/Black and White/Asian correlated strongly, but no more strongly than the D and OB correlations between White/Black and Flower/Insect. These results show that significant components of responses on indirect measures reflect domain-general cognitive skills that not only are not associative in nature, but are not even specifically relevant to the attitude object in question (see also, Mierke and Klauer, 2001, 2003; McFarland and Crouch, 2002; Calanchini and Sherman, 2013). As we shall see below, these “non-attitudinal” processes sometimes help to explain or even largely explain observed differences in implicit evaluations across people and contexts, and in response to interventions.

#### What Accounts for Interpersonal Variability?

Much work on implicit evaluation has examined differences among groups of people based on group membership, individual differences, etc. In standard analyses, any observed differences can only be explained by the operation of automatic associative processes: The groups in question must possess different evaluative associations. However, using the Quad model (Conrey et al., 2005; Sherman et al., 2008), we have shown that sometimes these differences are based on detection of correct responses (D) and overcoming bias (OB), neither of which are associative or entirely automatic. For example, those with high internal and low external motivation to respond without prejudice demonstrate less pro-White evaluative bias on the IAT. Modeling showed that these motivations are associated with a greater likelihood of D while performing the task (Gonsalkorale et al., 2011). As alluded to above, increased implicit evaluative bias with aging seems to be driven largely by diminished OB associated with aging, rather than differences in evaluative associations (Gonsalkorale et al., 2009a, 2014).

#### What Accounts for Contextual Malleability?

Another central focus of work on implicit evaluations concerns the extent to which they vary across contexts or can be changed by interventions. Again, in standard analyses, any observed effects must be explained by reference to changes in automatic associative responses. However, here, too, we have observed the critical roles of D and OB. For example, the implicit evaluative bias-reducing effects of counter-prejudicial training are associated with increases in D (Calanchini et al., 2013; see also, Rees et al., 2018). The reduction in evaluative bias associated with framing outgroup members in positive contexts is associated primarily with increased OB (Allen et al., 2010). In other work, we have applied a model of the Stereotype Misperception task (SMT; Krieglmeyer and Sherman, 2012) to estimate the prevalence of stereotype activation, an associative process (SAC), and stereotype correction (SAP), a non-associative process. In one study, we showed that reductions in implicit stereotyping associated with the formation of implementation intentions to respond without bias were associated with increased stereotype correction (Rees et al., 2019). Other work showed that increased implicit stereotyping associated with short response deadlines was associated with decreases in stereotype correction but not increases in stereotype activation (Rivers et al., 2020b). Yet another SMT study showed that the increase in implicit stereotyping associated with category salience is related more strongly to decreases in stereotype correction than increases in stereotype activation (Rees et al., 2020).

#### What Aspects of Implicit Evaluations Predict Behavior?

Other work shows the potential benefits of modeling for understanding when and why implicit evaluations predict behavior. In one study that applied the Quad model (Gonsalkorale et al., 2009b), the extent to which a Muslim confederate liked White non-Muslim interaction partners was based on the degree to which the interaction partners had exhibited both negative Muslim association activation (AC) and OB in performing an anti-Muslim Go/No Go task (e.g., Nosek and Banaji, 2001). Specifically, when the White interaction partners had low levels of AC on the task, the extent of the confederate’s liking was unrelated to the partner’s OB on the task. However, interaction participants with high levels of AC were liked to the extent that they had high OB estimates. Thus, the ability to overcome negative associations predicted the quality of the social interaction when those associations were strong. It is not merely a matter of the strength of evaluative bias. Standard analyses are unable to identify such interactions among processes.

## Conclusion

In this article, we addressed four fundamental conceptual and methodological problems that have undermined the implicit attitude research agenda. These four problems all originate in the framing of implicit and explicit evaluations and measures in terms of dual process models of cognition. One main takeaway is that conclusions about the constructs measured, the processes that influence their measurement, and the conditions under which those processes operate require independent assessment. Long-standing assumptions about the relationships among these variables threaten our ability to understand what implicit evaluations are and when, why, and how they affect social cognition and behavior. A firm grasp of these issues is critical for addressing questions such as who has implicit evaluative bias and why, when implicit evaluations are most likely to be problematic, and how can we best diminish problematic implicit evaluations and their influence on behavior.

Formal modeling techniques are a powerful way to better understand the nature of implicit evaluation and the processes that contribute to it. One important contribution of modeling has been the recognition that significant components of implicit evaluation have nothing to do with underlying mental associations or strictly automatic processes. A significant implication is that efforts to change implicit evaluations and their impact need not focus solely on efforts to change underlying associations. Rather, effective interventions may instead target self-regulatory and propositional processes that reduce the impact of evaluative associations without necessarily changing them (e.g., Rivers et al., 2020a).

## Author Contributions

Both authors listed have made a substantial, direct and intellectual contribution to the work, and approved it for publication.

## Conflict of Interest

The authors declare that the research was conducted in the absence of any commercial or financial relationships that could be construed as a potential conflict of interest.
